# Meteorological Register

**Published:** 1833-10

**Authors:** 


### METEOROLOGICAL REGISTER,

FROM JUNE 1 TO AUGUST 31,

By Messrs. HARRIS and Co., Mathematical Instrument Makers, 50, High Holborn.

| | | Thermometer. | | Barometer. | | De Luc's Hygrometer. | | Winds. | | Atmospheric Variation. |
|---|---|---|---|---|---|---|---|---|---|---|
| | | max. | min. | max. | min. | max. | min. | | | |
| June | 1 to 7 | 74 | 52 | 29.82 | 29.37 | 59 | 62 | WSW | SSW | fine & cloudy, little rain |
| | 14 | 75 | 53 | 30.04 | 29.30 | 65 | 57 | NW | WSW | — — — |
| | 21 | 72 | 48 | 29.87 | .43 | 68 | 59 | W | NNW | — — — |
| | 28 | 75 | 46 | .74 | .38 | 65 | 58 | NW | WSW | showery, rain, and cloudy |
| July | 5 | 70 | 49 | .91 | .60 | 62 | 57 | WSW | WNW | fine, and cloudy |
| | 12 | 73 | 56 | .85 | .44 | 69 | 50 | NNE | SE | — rain, and cloudy |
| | 19 | 79 | 54 | .96 | .51 | 65 | 59 | SW | NNE | — and cloudy |
| | 26 | 68 | 52 | 30.03 | .43 | 67 | 60 | NW | W | — — |
| Augu | 2 | 79 | 54 | .15 | 30.08 | 64 | 62 | NE | ESE | — — |
| | 9 | 66 | 50 | .10 | 29.81 | 63 | 58 | ENE | ESE | — little rain |
| | 16 | 70 | 51 | 29.92 | .40 | 63 | 58 | NW | NE | — cloudy |
| | 23 | 72 | 52 | .71 | .50 | 65 | 57 | W | NW | — fine |
| | 31 | 77 | 48 | 30.02 | 28.77 | 68 | 59 | NW | E | fine, and rain |

The quantity of Rain fallen from 1st June to 31st August—4 inches and 87-100ths.

